# Atrial Septal Defect: Larger Right Ventricular Dimensions and Atrial Volumes as Early as in the First Month After Birth—a Case–Control Study Including 716 Neonates

**DOI:** 10.1007/s00246-023-03211-z

**Published:** 2023-06-27

**Authors:** Anna Maria Dehn, Sofie Dannesbo, Anna Sellmer, Line Høffner, Elisabeth Blixenkrone-Møller, Anne-Sophie Sillesen, Anna Axelsson Raja, Niels Vejlstrup, Kasper Iversen, Henning Bundgaard, Vibeke Hjortdal

**Affiliations:** 1 Department of Cardiothoracic Surgery, Rigshospitalet, Copenhagen University Hospital, Blegdamsvej 9, 2100 Copenhagen, Denmark; 2 Department of Cardiology, Herlev-Gentofte Hospital, Copenhagen University Hospital, Copenhagen, Denmark; 3 Department of Cardiology, Rigshospitalet, Copenhagen University Hospital, Copenhagen, Denmark

**Keywords:** Atrial Septal Defect, Echocardiography, Neonates, Copenhagen Baby Heart Study, Right ventricular dimensions

## Abstract

Atrial septal defect (ASD) is characterized by a left-to-right shunt causing dilatation of the right atrium and right ventricle as well as pulmonary hyperperfusion. The detection of ASDs often occurs late in childhood or adulthood. Little is known about cardiac structure and function in neonates with ASD.

We analyzed neonatal echocardiograms from the Copenhagen Baby Heart Study, a multicenter, population-based cohort study of 27,595 neonates. We included 716 neonates with secundum-type ASDs and matched them 1:1 on sex and age at examination with neonates without ASD from the same birth cohort. Neonates with an ASD (median age 11 days, 52% female) had larger right ventricular (RV) dimensions than matched controls (RV longitudinal dimension end-diastole: 27.7 mm vs. 26.7 mm, *p* < 0.001; RV basal dimension end-diastole: 14.9 mm vs. 13.8 mm, *p* < 0.001; and RV outflow tract diameter 13.6 mm vs. 12.4 mm, *p* < 0.001). Atrial volumes were larger in neonates with ASD compared to controls (right atrial end-systolic volume: 2.9 ml vs. 2.1 ml, *p* < 0.001; and left atrial end-systolic volume 2.0 ml vs. 1.8 ml, *p* < 0.001). Tricuspid annular plane systolic excursion was larger in neonates with ASD than in controls (10.2 mm vs. 9.6 mm, *p* < 0.001). Left ventricular dimensions and function did not differ between neonates with ASD and controls. In conclusion, ASDs were associated with altered cardiac dimensions already in the neonatal period, with larger right ventricular dimensions and larger atrial volumes at echocardiography within the first 30 days after birth.

**ClinicalTrials.gov Identifier:** NCT02753348 (April 27, 2016).

**Supplementary Information:**

The online version contains supplementary material available at 10.1007/s00246-023-03211-z.

## Introduction

Atrial septal defects (ASD) are among the most common congenital heart defects. The morphological defect in the atrial septum will in most cases lead to a left-to-right shunt, due to higher pressure in the left atrium. The shunt results in an increased volume load on the right side of the heart. By the time of diagnosis, several other morphological changes in the heart are frequently found. Right ventricular dilatation and right atrial enlargement are among the most common changes [1]. Due to the natural history and clinical presentation, the diagnosis of a clinically significant ASD often does not occur until late in childhood or even adulthood [2]. The decision on whether and when to close a defect depends on several factors such as symptoms, the occurrence of paradoxical embolism, the ratio of pulmonary to systemic blood flow (Qp:Qs), and whether right-sided chambers are dilated, although the available evidence is sparse [3, 4].

Little is known about cardiac structure very early in life in patients with ASD since symptoms and diagnosis often occur later [5]. In utero, all fetuses have an interatrial communication, the foramen ovale, which allows for oxygenated blood from the placenta to be shunted from right to left, bypassing the pulmonary circulation. After birth, the expansion of the lungs and the decrease in pulmonary vascular resistance lead to increased pulmonary blood flow and rising pressure in the left atrium. The pressure changes cause the septum primum to cover the foramen ovale like a flap, closing the interatrial communication [6]. In neonates with ASD, however, these physiological changes in the cardiovascular system will reverse the shunt to left to right across the atrial septum. This transition occurs after birth, but it is unclear how rapidly the shunting affects the right-sided chambers and leads to structural changes.

This study aims to investigate if cardiac morphology and function in patients with ASD are different from matched controls as early as within the first month after birth. We explore this topic by examining an unselected cohort of neonates with ASD found in a large echocardiographic screening study of neonates.

## Material and Methods

We conducted a case–control study of echocardiographic parameters in neonates with ASD included in the Copenhagen Baby Heart Study.

### Copenhagen Baby Heart Study

The Copenhagen Baby Heart Study (CBHS) is a multicenter, population-based cohort study with prenatal inclusion. All expectant parents in the Capital Region of Denmark were offered inclusion in the study in the period April 1^st^, 2016, to October 31^st^, 2018 [7]. A total of 27,595 neonates were included in the study, whereof 25,590 underwent transthoracic echocardiography within the first 60 days after birth. Information on maternal and infant characteristics was retrieved from medical records and health registries and stored in the CBHS database [8]. The CBHS study cohort is intended to undergo lifelong follow-up.

### Acquisition of Neonatal Echocardiograms

In the CBHS, the neonatal echocardiographies were performed by sonographers and specialists trained in pediatric echocardiography using Vivid E9 ultrasound equipment (GE Vingmed Ultrasound, Horten, Norway). The CBHS echocardiographic protocol was established in accordance with the American Society of Echocardiography’s guidelines for pediatric echocardiography [9] and has been described in detail previously [7]. Cardiac sector transducers 12S-D and 6S-D were used to obtain standard subxiphoid, apical, left parasternal, and suprasternal views.

Parents who had given consent to be included in CBHS were reminded by text message after giving birth to a live-born infant and booked an appointment for the echocardiographic examination using a secure website. Neonatal echocardiograms in CBHS were performed while the infants were calm or sleeping.

### Analysis of Echocardiograms for Interatrial Communications

After the inclusion period, echocardiograms were reviewed offline using EchoPAC clinical software (version 113, GE Vingmed Ultrasound, Horten, Norway). To determine whether neonates had an ASD, two investigators systematically analyzed subxiphoid echocardiographic images of the atrial septum using a novel algorithm for the echocardiographic assessment of interatrial communications that was developed and validated in the CBHS and was recently published [10]. Categorization of interatrial communications was based on several echocardiographic criteria: We defined neonates as having an ASD in cases when color Doppler flow was crossing the atrial septum and when cross-sectional images showed either multiple visible communications (fenestrated ASD) or a single-visible communication with either a diameter of ≥ 4 mm or a location in the inferior part of the atrial septum. For interatrial communications with one single-visible communication on cross-sectional images, the diameter of the defect was measured.

### Study Cohort

Neonates with ASD were matched 1:1 with controls from the CBHS study cohort on sex and age at the time of echocardiographic examination. We excluded neonates with syndromes, chromosomal abnormalities, or other congenital heart disease.

### Measurement of Echocardiographic Parameters

For this study, we chose to investigate echocardiographic parameters of particular interest in patients with ASD. Namely, we investigated right ventricular (RV) dimensions (RV longitudinal dimension end-diastole; RV basal dimension end-diastole, and RV outflow tract (RVOT) diameter), tricuspid annular plane systolic excursion (TAPSE), atrial volumes (right atrial end-systolic volume (RAESV); left atrial end-systolic volume (LAESV)), left ventricular dimensions (left ventricular internal diameter end-diastole (LVIDd); left ventricular internal diameter end-systole (LVIDs); left ventricular posterior wall end-systole (LVPWs); left ventricular outflow tract(LVOT); interventricular septum end-diastole (IVSd)) as well as diameters for the inferior vena cava (IVC), and the main pulmonary artery (MPA). We also measured left ventricular ejection fraction (LVEF) and fractional shortening (FS). Measurements and analyses of echocardiographic parameters were done in accordance with the Recommendations for Quantification Methods During the Performance of a Pediatric Echocardiogram from the American Society of Echocardiography [11]. For the assessment of right ventricular dimensions that were not elaborately described in pediatric guidelines, we followed the Guidelines for the Echocardiographic Assessment of the Right Heart in Adults from the American Society of Echocardiography [12]. For details on echocardiographic parameters, see Supplementary Table 4.

Analyses and measurements of echocardiographic outcome parameters were made by two investigators blinded to the presence of ASD, as they did not assess images of the atrial septum with Color Doppler. Echocardiograms from neonates with ASD and matched controls were randomly distributed between the two investigators. Echocardiographic analyses were frequently supervised by a third investigator to minimize the risk of inter- and intraobserver variability.

### Statistical Analyses

Continuous variables are presented as mean values (SD) or median values (IQR), where appropriate. Categorical variables are presented as absolute numbers (percentages). Comparison between cases (neonates with ASD) and matched controls was performed using Student’s t test. Bonferroni’s correction for multiple testing was applied, and *p* values < 0.003 were considered statistically significant (analyses for 15 echocardiographic parameters: *p* 0.05 / 15 = 0.003).

We performed subgroup analyses on the influence of the size of the ASD, the neonates’ age at examination, and the influence of fenestrated defects vs. single defects. For analyses on the influence size of the ASD, we looked further into the subgroup where the ASD diameter measurement was feasible. We plotted the echocardiographic outcome variables that showed a difference in means between neonates with ASD and controls in the main analysis against the ASD diameters to identify potential associations and applied a linear regression model with a 95% confidence interval. For subgroup analyses on the neonates’ age at examination, we compared neonates examined at age 0–7 days old with neonates examined at age 8–30 days old. For analyses on the influence of fenestrated defects, we performed analyses on a subgroup of neonates with ASD, who had complete data on all RV parameters and atrial volumes. We compared the echocardiographic outcome variables that differed between neonates with ASD and controls in the main analysis using Student’s t test with Bonferroni’s correction for multiple testing.

Statistical analyses were performed using R statistical software v. 1.2.1335 (Boston, MA, USA).

## Results

### Study Cohort

We included 716 neonates with secundum-type ASD (52% female, mean age at examination 11 days) and 716 controls matched on sex and age at examination. Descriptive characteristics are shown in Table 1. Neonates with ASD and controls were comparable with regard to gestational age at birth, birth weight and length, and body surface area.

**Table 1:** Descriptive characteristics for neonates with ASD and matched controls

	Newborns with ASD (*n* = 716)	Controls (*n* = 716)
*Newborn characteristics*		
Sex, female, *n*(%) *	370 (52%)	370 (52%)
Gestational age at birth (days), median (IQR)	280 (272–287)	281 (273–287)
Birth weight (g), mean (SD)	3474 (524)	3488 (504)
Birth length (cm), mean (SD)	51.3 (2.4)	51.6 (2.2)
Age at examination (days), median (IQR)*	11 (6–14)	11 (6–14)
BSA (Haycock formula), mean (SD)	0.23 (0.02)	0.23 (0.02)
*Premature*		
Gestational age at birth <37 weeks, *n* (%) Gestational age at birth <32 weeks, *n* (%)	44 (6%)	0 (0%)
	1 (0%)	33 (5%)
*Maternal characteristics*		
Maternal age at birth (years), median (IQR)	31 (29–35)	32 (28–35)
Maternal pre-pregnancy BMI (kg/m^2^), mean (SD)	23.9 (4.6)	23.7 (4.5)

* = matching criteria. Categorical variables are displayed as absolute numbers (percentages) and continuous variables as either median values (interquartile ranges) or mean values (standard deviation). Abbreviations; ASD, atrial septal defect; BMI, body mass index; BSA, body surface area

The direction of shunting in neonates with ASD was left-to-right in the majority of cases (*n* = 644; 90%), while a smaller proportion of neonates had an additional component of right-to-left shunting (*n* = 72; 10%). None of the neonates had exclusively right-to-left shunting.

**Table 2:** Echocardiographic characteristics for neonates with ASD and matched controls

Echocardiographic measurements		Unit	Neonates with ASD *n* = 716		Controls *n* = 716		*P* value
			mean (SD)	*n*	mean (SD)	*n*	
RV dimensions and function	RV longitudinal dimension end-diastole	mm	27.7 (4.3)	563	26.7 (3.8)	535	** < 0.001**
	RV basal dimension end-diastole	mm	14.9 (2.2)	558	13.8 (2.6)	535	** < 0.001**
	RVOT diameter	mm	13.6 (2.5)	591	12.4 (2.1)	579	** < 0.001**
	TAPSE	mm	10.2 (1.8)	674	9.6 (1.9)	681	** < 0.001**
LV dimensions and function	LVIDd	mm	19.9 (1.7)	684	19.9 (1.6)	669	0.62
	LVIDs	mm	13.7 (1.4)	683	13.5 (1.3)	659	0.09
	LVPWd	mm	2.0 (0.4)	682	2.0 (0.4)	664	0.38
	LVOT diameter	mm	7.2 (0.9)	673	7.3 (0.9)	663	0.005
	IVSd	mm	2.5 (0.5)	682	2.5 (0.4)	665	0.96
	LVEF	%	62.7 (5.1)	683	62.1 (5.4)	659	0.03
	LV FS	%	31.4 (3.6)	683	31.8 (3.9)	659	0.03
Atrial volumes	LAESV	ml	2.0 (0.7)	596	1.8 (0.7)	590	** < 0.001**
	RAESV	ml	2.9 (1.1)	370	2.1 (0.8)	400	** < 0.001**
Other	IVC diameter	mm	3.9 (1.2)	634	3.9 (0.9)	634	0.13
	MPA diameter	mm	9.0 (1.6)	563	8.7 (1.4)	570	0.005

*p* value < 0.003 is considered statistically significant (after Bonferroni correction) and is depicted in bold

*IVC* inferior vena cava, *IVSd* Interventricular septum end-diastole, *LAESV* left atrial end-systolic volume, *LV* left ventricle, *LVEF* left ventricular ejection fraction, *LV FS* left ventricular fractional shortening, *LVIDd* Left ventricular internal dimension end-diastole, *LVIDs* Left ventricular internal dimension end-systole, *LVOT* left ventricular outflow tract, *LVPWd* Left ventricular posterior wall thickness end-diastole, *MPA* main pulmonary artery, *RAESV* right atrial end-systolic volume, *RV* right ventricle, *RVOT* right ventricular outflow tract, *TAPSE* tricuspid annular plane systolic excursion

### Echocardiographic Parameters

Neonates with ASDs had larger RV basal diameter at end-diastole than controls as well as larger RV longitudinal dimensions. End-systolic atrial volumes were larger in neonates with ASD than in controls for both the right atrium and left atrium. Right ventricular outflow tract diameter was larger in neonates with ASD than in controls, while MPA diameter was slightly larger but did not reach statistical significance after Bonferroni correction. The tricuspid annular plane systolic excursion was larger in neonates with ASD compared to controls. Left ventricular dimensions, LV function, the IVSd, and LVOT diameter did not differ between neonates with ASD and controls; neither did the diameter of IVC at expiration. Echocardiographic characteristics for neonates with ASD and matched controls are shown in Table 2.

### Influence of the Size of the ASD

Of the 716 neonates with ASD, measurement of the septal defect diameter was feasible in 348 neonates with regard to the used algorithm for the assessment of interatrial communications [10]. The mean diameter of the defects was 4.4 mm (range 3.5–10.5 mm).

Echocardiographic values for parameters that differed between neonates with ASD and controls (RV longitudinal dimension end-diastole, RV basal dimension end-diastole, RVOT, TAPSE, LAESV, and RAESV) were plotted against the size of the ASD and are shown in Supplementary Fig. 2. No clear correlation between the size of the defect and the increase in right ventricular dimensions or atrial volumes was seen. However, when fitting linear regression models to the plots, a slight positive association between the diameter of ASD and RAESV was found (p = 0.02). TAPSE also increased slightly with increasing diameter of ASD (p = 0.01).

### Influence of the Neonatal Age at Examination

To investigate whether differences in echocardiographic parameters between neonates with and without ASD were present at birth or developed during the first month of life, we divided the cohort into two subgroups based on the neonates’ age at examination. Specifically, we compared neonates with ASD examined during the first week after birth (0 to 7 days old) with neonates from the control group examined at the same age. Likewise, we compared neonates with ASD examined at age 8 to 30 days with controls (Table 3).

**Table 3:** Echocardiographic characteristics for age-differentiated subgroups: age 0–7 days and 8–30 days at examination

Echocardiographic measurements			Age at examination 0–7 days *n* = 454			Age at examination 8–30 days *n* = 978		
			Neonates with ASD (*n* = 228)	Controls (*n* = 226)	*p*-value	Neonates with ASD (*n* = 488)	Controls (*n* = 490)	*p*-value
RV dimensions and function	RV longitudinal dimension end-diastole	mm	25.6 (4.2)	26.7 (3.5)	0.01	28.7 (4.0)	26.7 (3.9)	** < 0.001**
	RV basal dimension end-diastole	mm	15.1 (2.3)	13.9 (2.7)	** < 0.001**	14.9 (2.2)	13.8 (2.6)	** < 0.001**
	RVOT diameter	mm	1.4 (2.6)	12.5 (2.1)	** < 0.001**	13.5 (2.4)	12.5 (2.1)	** < 0.001**
	TAPSE	mm	9.7 (1.7)	8.6 (1.7)	** < 0.001**	1.1 (1.8)	1.0 (1.8)	** < 0.001**
Atrial volumes	LAESV	ml	1.8 (0.7)	1.6 (0.6)	** < 0.001**	2.1 (0.6)	1.9 (0.7)	**0.0019**
	RAESV	ml	3.0 (1.1)	1.9 (0.8)	** < 0.001**	2.8 (1.1)	2.1 (0.8)	** < 0.001**
LV dimensions and function	LVOT diameter	mm	7.1 (0.9)	6.9 (0.9)	0.003	7.4 (0.9)	7.4 (0.9)	0.15
	LVIDd	mm	19.4 (1.7)	19.4 (1.5)	0.914	20.1 (1.7)	20.0 (1.7)	0.49
	LVIDs	mm	13.2 (1.3)	13.0 (1.2)	0.06	13.9 (1.3)	13.8 (1.3)	0.35
	LVPWd	mm	2.0 (0.5)	2.0 (0.4)	0.39	2.1 (0.5)	2.1 (0.4)	0.10
	IVSd	mm	2.4 (0.5)	2.4 (0.4)	0.98	2.5 (0.5)	2.5 (0.4)	0.99
	LVEF	%	63.3 (5.0)	65.0 (5.9)	0.004	61.5 (5.0)	61.8 (4.8)	0.54
	LV FS	%	32.1 (3.6)	33.3 (4.5)	0.003	31.0 (3.6)	31.1 (3.4)	0.62
Other	IVC diameter	mm	4.0 (1.5)	3.8 (1.0)	0.25	3.9 (1.0)	3.9 (0.9)	0.34
	MPA diameter	mm	9.0 (0.2)	8.9 (0.2)	0.25	9.0 (0.2)	9.0 (0.1)	0.007

*P*-value < 0.003 is considered statistically significant (after Bonferroni correction) and is depicted in bold

*IVC* inferior vena cava, *IVSd* Interventricular septum end-diastole, *LAESV* left atrial end-systolic volume, *LVEF* let ventricular ejection fraction, *LF FS* left ventricular fractional shortening, *LVIDd* Left ventricular internal dimension end-diastole, *LVIDs* Left ventricular internal dimension end-systole, *LVOT* left ventricular outflow tract, *LVPWd* Left ventricular posterior wall thickness end-diastole, *MPA* main pulmonary artery, *RAESV* right atrial end-systolic volume, *RV* right ventricle, *RVOT* right ventricular outflow tract, *TAPSE* tricuspid annular plane systolic excursion

For the echocardiographic parameters that showed differences in means between neonates with ASD and controls in the main analyses, the majority also differed between groups in this subgroup analysis at either age at examination. The exception from this was RV longitudinal dimension end-diastole which did not differ between cases and controls during the first week after birth.

### Influence of Fenestrated Defects vs. Single Defects

We found no differences in right ventricular dimensions or atrial volumes between neonates with single defects (*n* = 139) and neonates with fenestrated defects (*n* = 134) apart from a slightly larger RAESV in neonates with single defects compared to neonates with fenestrated defects (2.9 ml. vs. 2.6 ml, *p* = 0.006; *p* < 0.008 considered statically significant after Bonferroni correction; data not shown).

## Discussion

This is the largest study to date examining cardiac structure and function in neonates with secundum-type ASD. Neonates with ASD exhibited larger dimensions of the RV, the RVOT, and both atria as early as within the first month after birth. We demonstrated this through echocardiography in a cohort of 716 neonates with ASD, as compared to a control group of 716 neonates without ASD.

The dimensions of the right heart chambers are included in the evaluation of the hemodynamic significance of an ASD. These structural changes are considered secondary to the ASD and the changes in blood flow, but it remains unknown at which timepoint the pathophysiological changes occur [13, 14]. Our results indicate that the morphological changes occur very early in the neonate, after only a few days of shunting over the ASD.

In the fetal circulation, both the physiological foramen ovale and the abnormal ASD allow for right-to-left shunting. Hence, in utero, there should be no significant differences in blood flow between fetuses with the physiological foramen ovale and fetuses with an ASD. At the transition to postnatal life, the pulmonary circulation opens, with a significant reduction in pulmonary vascular resistance and a redirection of blood flow through the lungs and back into the left atrium. Normally, the primum septum acting as a flap will close the interatrial communication, and the circulation will continue as a parallel circulation with almost equal volumes on the left and the right sides. In the situation of an ASD, however, a defect remains, through which the right atrium will receive a larger volume of blood. The immediate effect of an increased blood flow and volume load in a compliant system will be dilatation. In our subgroup analyses, we looked specifically at neonates with an ASD aged 0–7 days at the time of echocardiographic examination and found likewise the right atrium and RV to be larger than in matched controls. These results emphasize that only a few days of left-to-right shunting over the ASD have an impact on the right-sided chambers.

In adults, the compliance of the ventricles decreases with increasing age [15, 16]. In the fetal heart, compliance is considerably lower than in adults, while in the neonatal heart, the compliance of the ventricles is higher than in fetal state, yet lower than in adults. The RV has a higher compliance than the LV regardless of age [17]. A possible explanation for the lower compliance in the neonatal heart might be a relatively high amount of collagen, especially type I collagen, mainly providing rigidity [18]. As a result of the relatively low compliance, the intraventricular pressure and wall tension will rise comparably fast in response to volume overload in the neonatal heart. The left-to-right shunt in ASD causes a volume overload on the right side of the heart, and from our results, we can hypothesize that the resulting increase in pressure and wall tension will lead to morphological changes even in the presence of small shunts and even after only a few days of shunting.

The RVOT is recommended to be part of the assessment of the RV in adults [12] but normative data on infants and children are limited. In accordance with our findings, Koestenberger et al. showed RVOT diameter to be enlarged in children with secundum ASD (*n* = 115, age range 2 days–18 years) [19]. An increased pulmonary blood flow in patients with ASD will over time possibly lead to enlargement of the MPA as well. In our results, the MPA diameter was slightly larger in neonates with ASDs but did not reach statistical significance after Bonferroni correction.

TAPSE is a recommended tool for assessing right ventricular systolic function in standard echocardiographic exams [11], even though its clinical significance in pediatric patients is not well established. We found TAPSE to be larger in neonates with ASD than in controls although the mean values for both groups were within previously published normal ranges for TAPSE in neonates [20]. Koestenberger et al. investigated TAPSE in patients with unrepaired isolated secundum ASDs (*n* = 200, age range 0–21 years) and found no significant difference when compared to a control group [21]. In accordance with our findings, Arce et al. looked specifically at the longitudinal motion of the atrioventricular annuli in children including a group with right ventricular overload due to ASD (n = 25, age range 1–37 years) and found an exaggerated normal pattern of movement with a higher than normal absolute and percentual displacement of the tricuspid lateral ring [22].

We also found the left atrial volume to be larger in neonates with ASDs than in neonates without ASD. Left atrial enlargement in patients with ASD has previously been described as a consequence of the volume overload similar to right-sided enlargements [23]. An alternative pathophysiological hypothesis could be a primary dilatation of the atria in utero. In the stretched atrial wall, the septum primum may not sufficiently cover the foramen ovale, leading to a secundum-type ASD.

The dimensions and function of the LV, LVOT, and interventricular septum did not differ between neonates with ASD and controls, which is in accordance with earlier studies [24].

A limitation of all echocardiographic studies includes interobserver variability. For the present study, we sought to minimize interobserver variability by having the echocardiographic analyses performed by only two investigators, who had received equal training and a thorough introduction to the measurements and methods used for this study. Also, the investigators were blinded to whether the neonates had an ASD or not, by not accessing the images with color Doppler over the atrial septum. Repeatability and reproducibility of neonatal echocardiography in the CBHS have previously been tested, showing good inter- and intraobserver agreement for most measurements [25]. Potential limitations of the CBHS data and cohort have been discussed previously [7, 8]. Neonates born preterm were under-represented in the CBHS cohort, limiting the generalizability of results to preterm infants.

The CBHS is presenting robust echocardiographic data on the largest cohort of neonates to date. Neonatal echocardiograms of > 25,000 neonates were performed, using a TTE protocol in accordance with international standards. However, as the cohort was not specifically set up for the assessment of children with shunts, we have limited data on right-sided dimensions. Echocardiographic techniques are steadily being improved with advanced analyses being made possible by for example 3D echocardiography and strain imaging [26]. For optimal assessment of right ventricular dimensions, magnetic resonance imaging would also have been preferred since it is considered the gold standard and superior to echocardiography [27].

The neonates included in our study were not diagnosed with ASD due to symptoms but found this early after birth because they were part of a large population-based cohort study with echocardiographic examination. This unselected cohort of neonates with ASD is unique, allowing us to report echocardiographic characteristics in neonates with and without ASD from the same birth cohort.

In our subgroup analyses, we found a slight positive association between the diameter of the ASD and RAESV, and TAPSE, but no clear pattern for the other echocardiographic variables. Studies specifically on cohorts of patients with small ASDs show that morbidity and mortality are seen even in cases where the ASD is considered small and hemodynamically insignificant [28–31].

The cohort of neonates with ASD in the present study might include neonates where the defects are both asymptomatic and smaller than usually seen in clinical settings. The reported morphological differences between neonates with ASD and controls found in this study may be relatively small in absolute numbers but reached convincing statistical significance due to a large number of included subjects. However, the study design with the large cohort of neonates in the CBHS and the method with thorough and blinded echocardiographic analyses for this present study validate our findings and make up the strengths of our study. This new knowledge of early morphological changes contributes to our pathophysiological and anatomical understanding of ASDs.

## Conclusion

Neonates aged 0–30 days with ASDs had larger right ventricular dimensions and larger atrial volumes than matched controls. These findings indicate that the morphological cardiac changes typical for ASDs are found very early in life after only a few days of left-to-right shunting.

## Supplementary Information

Below is the link to the electronic supplementary material.

Supplementary file1 (DOCX 801 KB)

## Data Availability

The data that support the findings of this study are not publicly available due to the privacy of research participants. The data will be shared upon reasonable request to the corresponding author.
