# Supplementary material for: Atrial Septal Defect: Larger Right Ventricular Dimensions and Atrial Volumes as Early as in the First Month After Birth—a Case–Control Study Including 716 Neonates
Source: Pediatr Cardiol. 2023 Jun 27;44(7):1578–86. doi: 10.1007/s00246-023-03211-z (PMC10435647; doi:10.1007/s00246-023-03211-z)
Supplement: Supplementary file 1 — Supplementary file1 (DOCX 801 KB) [file 246_2023_3211_MOESM1_ESM.docx]

# Supplementary Material

**Supplementary Fig. 1** Diagnostic algorithm for classification of interatrial communications on transthoracic echocardiography in newborns [10]
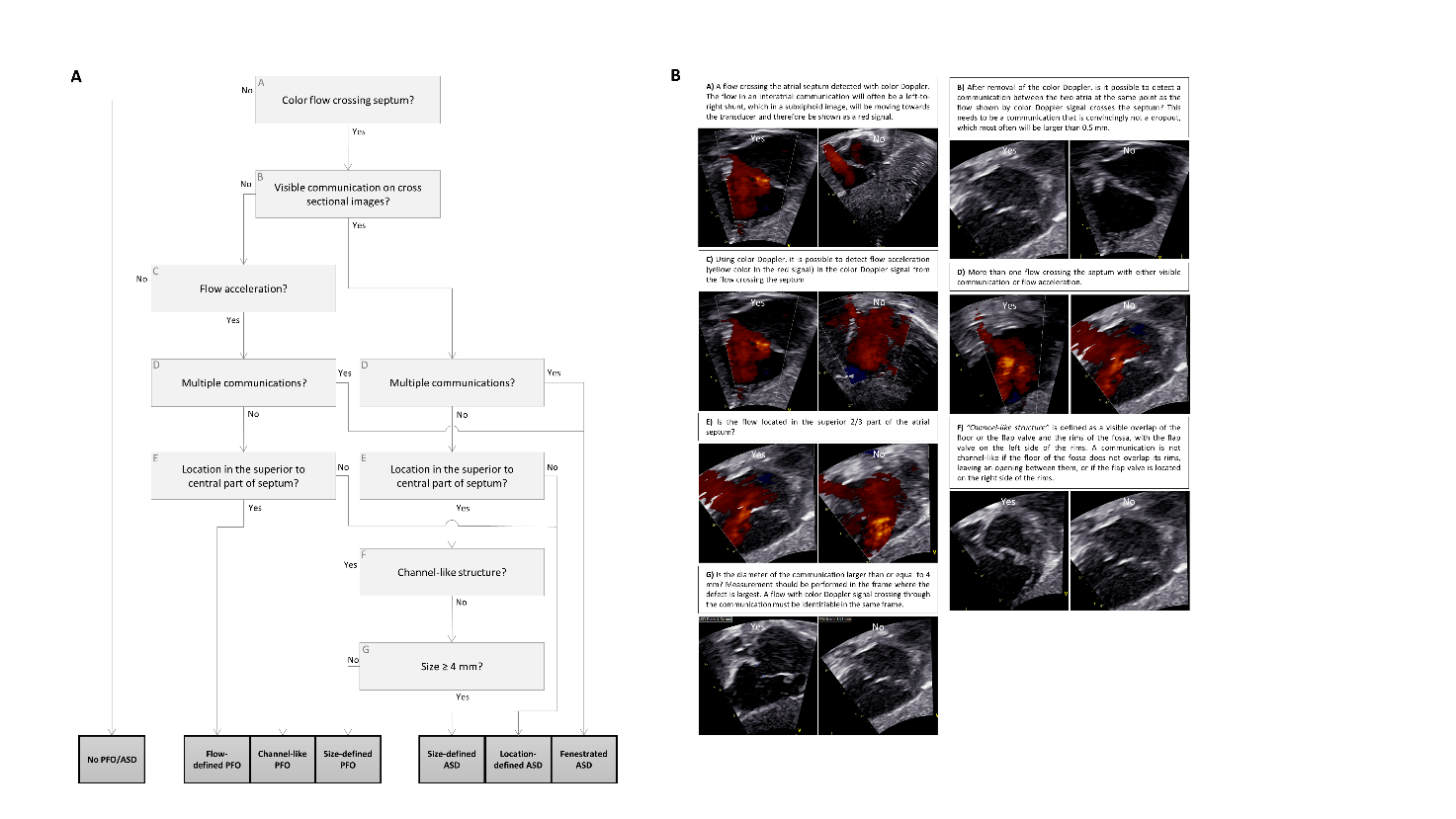


**Supplementary Table 4** Methods for acquisition and analyses of echocardiographic parameters

| **Measurement** | **View** | **Technique** | **Unit** | **Timing / Location** | **Description** |
| --- | --- | --- | --- | --- | --- |
| RV length | Apical 4-chamber, RV centered* | cross-sectional | cm | Ventricular end-diastole | RV longitudinal dimension measured from RV basal border (defined as the line connecting the tricuspid valve annular hinge points) to RV apex |
| RV basal diameter | Apical 4-chamber, RV centered* | cross-sectional | cm | Ventricular end-diastole | RV maximal short axis dimension in the basal one-third of the RV, below the tricuspid valve annular hinge points |
| RVOT diameter | Left parasternal short-axis, aortic valve level | cross-sectional | cm | Ventricular end-diastole | RVOT linear dimension measured from the anterior aortic wall to the RV free wall above the aortic valve |
| TAPSE | Apical 4-chamber, RV centered | M-Mode | cm | Cursor through tricuspid annulus | Longitudinal motion of the tricuspid annulus |
| LAESV | Apical 4-chamber, LV centered | cross-sectional | ml | End-systole | Tracing of left atrial borders to obtain LA planimetered area, and subsequent calculation of volume |
| RAESV | Apical 4-chamber, RV centered* | cross-sectional | ml | End-systole | Tracing of right atrial borders to obtain RA planimetered area, and subsequent calculation of volume |
| LVOT diameter | Left parasternal long-axis, focus on aorta | cross-sectional | cm | Mid-systole | Maximum dimension of the narrowest subvalvar left ventricular outflow tract |
| IVC diameter | Subxiphoid, abdominal long axis | cross-sectional | mm | Expiration | Maximum diameter of inferior vena cava just before the opening of the hepatic vein into the IVC |
| MPA diameter | Left parasternal short-axis, pulmonary trunk level | cross-sectional | cm | Mid-systole | Diameter of the main pulmonary artery between the pulmonary sinotubular junction and bifurcation |
| LVIDd | Left parasternal long-axis | cross-sectional | cm | Ventricular end-diastole | Left ventricular internal dimension measured just below mitral annulus |
| LVIDs | Left parasternal long-axis | cross-sectional | cm | Ventricular end-systole | Left ventricular internal dimension measured just below mitral annulus |
| LVPWd | Left parasternal long-axis | cross-sectional | mm | Ventricular end-diastole | Left ventricular posterior wall thickness measured just below mitral annulus |
| IVSd | Left parasternal long-axis | cross-sectional | mm | Ventricular end-diastole | Interventricular septal wall measured just below mitral annulus |
| LVEF | Left parasternal long-axis | cross-sectional | % | Systole | calculated by the GE Vivid 9 system using Teichholz’s formulae |
| LV FS | Left parasternal long-axis | cross-sectional | % | Systole | calculated by the GE Vivid 9 system using Teichholz’s formulae |

Abbreviations. IVC, inferior vena cava; IVSd, Interventricular septum end-diastole; LAESV, left atrial end-systolic volume; LVEF, left ventricular ejection fraction; LF FS, left ventricular fractional shortening; LVIDd, Left ventricular internal dimension end-diastole; LVIDs, Left ventricular internal dimension end-systole; LVOT, left ventricular outflow tract; LVPWd, Left ventricular posterior wall thickness end-diastole; MPA, main pulmonary artery; RAESV, right atrial end-systolic volume; RV, right ventricle; RVOT, right ventricular outflow tract; TAPSE, tricuspid annular plane systolic excursion. *If no RV-centered apical 4-chamber image of adequate quality was available, LV-centered apical 4-chamber image was used instead.

**Supplementary Fig. 2** Echocardiographic parameters by size of the ASD for A) RV length, cm; B) RV basal diameter, cm; C) RVOT diameter, cm; D) TAPSE, cm; E) LAESV, ml; F) RAESV, ml

The figure illustrates sub analyses on the influence of the size of the ASD. Plots depict echocardiographic measurements (y-axis) plotted against the diameter of the SD (x-axis). Linear regression models with 95% CI are fitted to the plots.

**
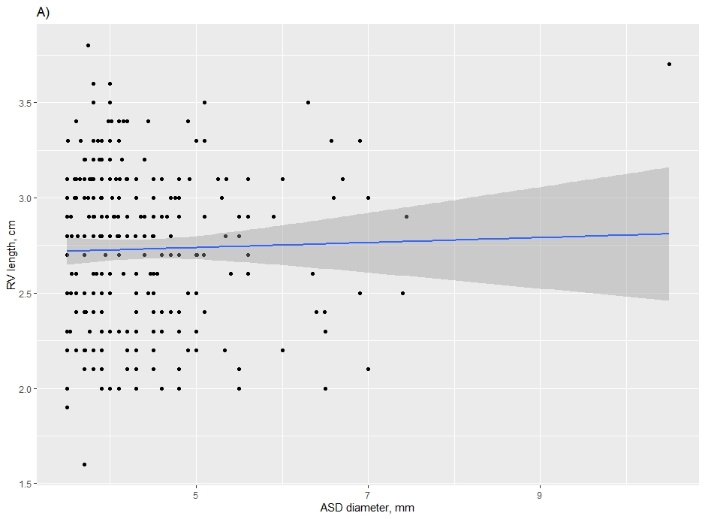
**
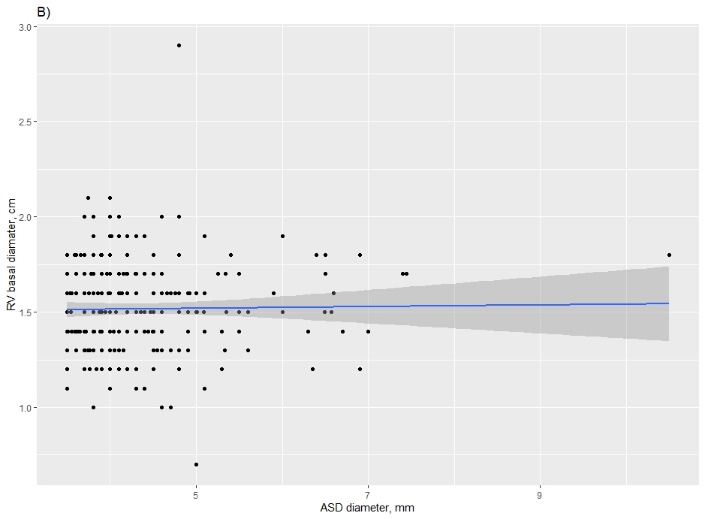

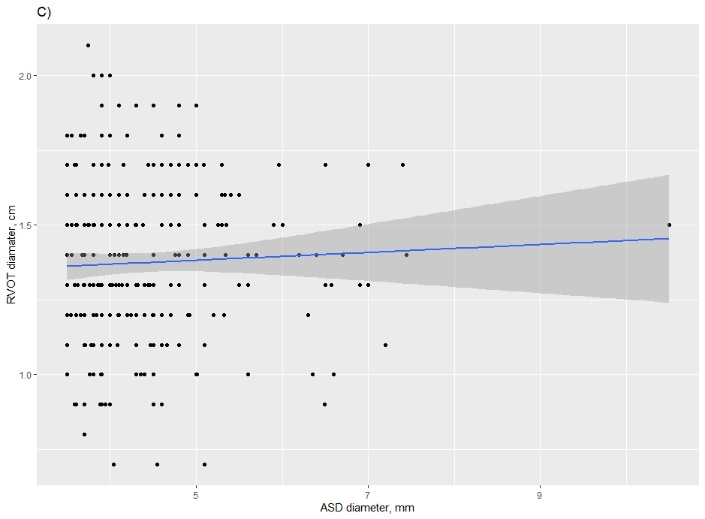

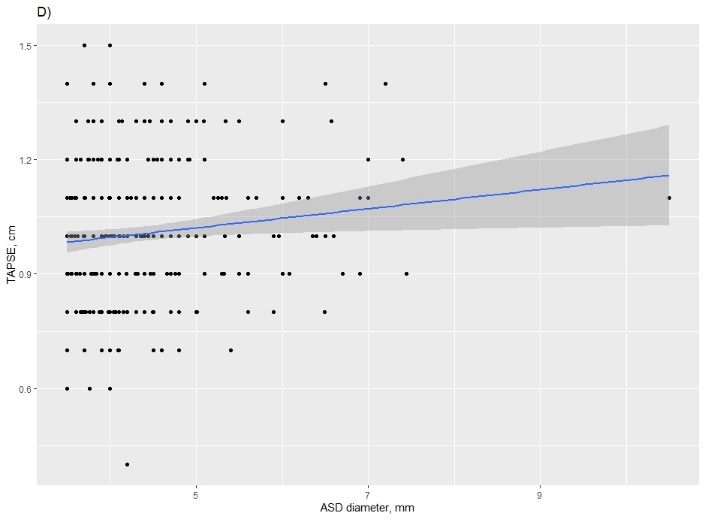

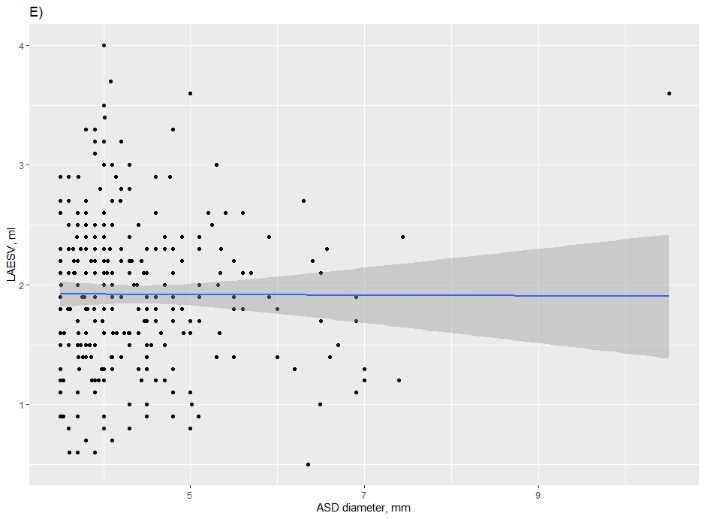

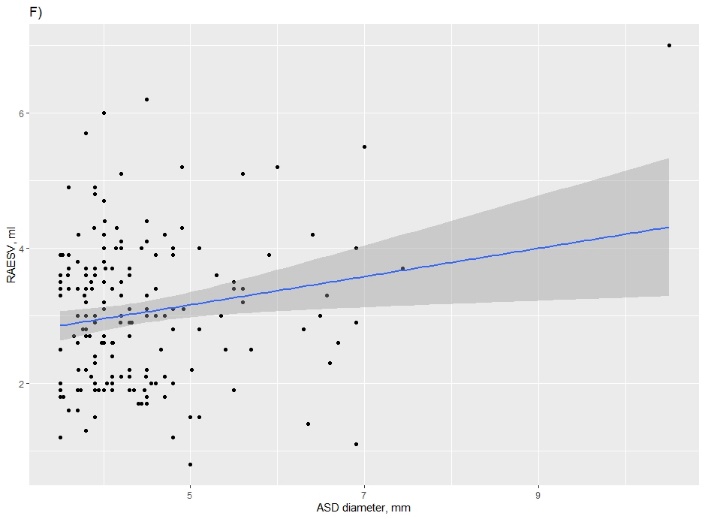


Abbreviations. ASD, atrial septal defect; LAESV, left atrial end-systolic volume; RAESV, right atrial end-systolic volume; RV, right ventricle; RVOT, right ventricular outflow tract; TAPSE, tricuspid annular plane systolic excursion.
